# Supplementary material for: A cortical–hippocampal communication undergoes rebalancing after new learning
Source: eLife. 2026 Oct 1;14:RP107370. doi: 10.7554/eLife.107370 (PMC13630386; doi:10.7554/eLife.107370)
Supplement: Peer review history

# Editor report: eLife Assessment

**DOI:** 10.7554/eLife.107370.3.sa0

**Authors:** Adrien Peyrache

**Affiliations:** McGill University Canada

**Keywords:** Convincing, Valuable

This **valuable** study investigates communication from the anterior cingulate cortex to the hippocampus during learning. The optogenetic evidence supporting the functional connection, including the interneurons likely to be involved, is **convincing**. However, the evidence linking this pathway to learning and memory and certain aspects of the statistical interpretation remains limited. Overall, the work will be of interest to neuroscientists studying cortical–hippocampal interactions and learning and memory.

# Referee report: Reviewer #2 (Public review)

**DOI:** 10.7554/eLife.107370.3.sa1

This work is composed of two largely independent parts. The first part (Figures 1-4) attempts to study correlations between the anterior cingulate cortex (ACC) and hippocampal area CA1 in the context of learning and memory; a number of issues including missing controls make this part inconclusive and hard to interpret. The second part (Figures 5 and 6) presents evidence for a pathway in which inputs from the ACC indirectly inhibit pyramidal cells in the superficial sublayer of CA1. The optogenetic evidence demonstrating the functional connection, including the interneurons likely to be involved, is convincing, making the second part of the manuscript a valuable contribution to neuroscience. However, I do not see evidence for this connection in the correlational analyses in the first part of the study, making the involvement of this pathway in learning and memory uncertain.

Strengths:

The biggest strength of the work is the optogenetic manipulation experiments in the second part of the study (Figures 5 and 6), which convincingly demonstrate that stimulation of ACC pyramidal neurons activates an interneuron population with symmetric spike waveforms, and inhibits parvalbumin interneurons and pyramidal cells in CA1sup, while CA1deep cells remained largely unaffected by the stimulation.

Weaknesses:

The main weakness is the disconnected nature of the two parts of the study. The second part convincingly shows that ACC provides a net inhibitory drive to the hippocampus (at least to CA1sup pyramidal and PV cells, while CA1deep cells were mostly unaffected). However, the first part investigates positive cross-correlations between pre-ripple ACC activity and subsequent CA1 ripple activity. This can be observed in Figure 1-supplement 1, where CA1 cells' activity peaks around 70ms after ACC spikes. Moreover, the GLM analyses were also based on positive ACC cell-CA1 cell pair correlations as the authors reported no bias towards negative weights for the GLM analyses (see the rebuttal letter). Thus, the correlational and GLM analyses in the first part primarily characterize a positive ACC-CA1 relationship, rather than the inhibitory influence demonstrated in the second part; the two parts of the manuscript therefore investigate different phenomena (possibly confounding inputs and network effects in part 1 versus the direct ACC-CA1 connection in part 2).

The key problem is that the main results of the two parts - namely, a dampening of the positive cross-correlations following learning in part 1 and the inhibitory ACC-CA1 connection revealed in part 2 - would be contradictory if they were interpreted as describing the same phenomenon. If the inhibitory ACC-CA1 connection was key to the downregulation of CA1 activity after learning as the authors suggest in the discussion, then we would expect ACC activity driving this change to be particularly predictive of CA1 activity in this post-learning period. Indeed, because prediction gain measures how well ACC spiking can predict subsequent CA1 spiking, any additional predictive information from the direct ACC->CA1 pathway should increase prediction gain. Instead, prediction gain decreased following learning. Thus, the positive (dampened after learning) ACC-CA1 correlations observed in the first part cannot be explained by the inhibitory ACC-CA1 pathway demonstrated in the second part. The most likely explanation is therefore that the cross-correlations studied in part 1 are dominated by other factors (such as shared inputs from other areas or coordination of cortical rhythms) and reported changes in prediction gain therefore primarily reflect changes in these factors, while the contribution of the direct ACC-CA1 pathway is drowned out and undetectable using this approach. As they stand, the two halves of the paper cannot be reconciled into the same framework.

The second weakness is the lack of control for learning. The main result of part 1 of the study is that there is dampening of the (positive) CA1 response to ACC pre-ripple activity after learning. However, nothing indicates this is due to learning as there is no control data with no learning. Moreover, the pre- and post- task periods were not matched for duration and sleep depth, so it is entirely possible that the observed dampening could be due to reduced recruitment of some cells in ripples. An appropriate control would therefore be important for attributing this to learning

The final weakness is statistical and goes beyond the lack of hierarchical statistics (which is also an issue with this work). The failure of a test to reach significance cannot be interpreted as evidence for the opposite. Yet the authors interpret it as such: for example, the lack of significant correlation between prediction gain values in pre- and post-task sleep in Figure 3C (p=0.14) is incorrectly interpreted as proof that ACC-CA1sup communication has reorganized as a result of learning. The claims of reorganization (mentioned multiple times in the abstract) hinge solely on this failed statistical test. Yet a failure to reach significance does not successfully demonstrate reorganization as it could result from a number of other reasons, including lack of statistical power or noisy estimates. To demonstrate reorganization, one would need to show that the observed change is greater than expected under an appropriate control (e.g. control task with no learning; or sleep data split in two halves), but this is missing from this manuscript.

Note that in the entire manuscript, the only differences between CA1sup and CA1deep are reported as two independent tests, one of which is significant and the other does not reach statistical significance. However, this is not evidence for different effects in CA1sup and CA1deep and statements like "we uncovered a pathway-specific difference" to describe these findings are unwarranted and not supported by the data; only direct statistical comparison between the two effects could support such claims. The exception to this weakness is the optogenetic experiments in Figure 5 where CA1sup and CA1deep responses to optogenetic ACC stimulation were directly compared and found to be different.

# Author comment: Author response

**DOI:** 10.7554/eLife.107370.3.sa2

**Authors:** Arron F Hall, Dong V Wang

**Affiliations:** Drexel University College of Medicine Philadelphia United States; Drexel University College of Medicine Philadelphia United States

The following is the authors’ response to the original reviews.

**Public Reviews:**

**Reviewer #1 (Public review):**

Summary:

This work by Hall et al provides a novel and important new finding about communication between the anterior cingulate cortex (ACC) and the CA1 region of the dorsal hippocampus: there is a clear ability of ACC to predict CA1 activity, and that is modulated by learning/experience. Furthermore, they have some evidence that the modulation differs by whether the CA1 neurons were in the deep versus superficial sub-layer of CA1. The evidence is suggestive of new and exciting findings, but some gaps and weaknesses remain to be addressed before I believe all of the authors' claims can be supported. The figures also need to be slightly better organized, and the discussion is missing a major dimension in my opinion. Overall, this is a strong submission, but with some gaps to fill.

Strengths:

(1) This is a well-written manuscript - the introduction was especially clear, well-cited, and motivating.

(2) The sub-layer specific communication between ACC and CA1 represents the discovery of a novel and functionally impactful piece of neurobiology.

(3) Optogenetics was an important verification of ACC-CA1 communication, as was the analysis of neurons by waveform type.

Weaknesses:

(1) Figure 2: Why are the data separated into two groups from the outset? If all data are combined, is there a general drop in prediction gain from pre to post?

Thank you for bringing this to our attention. In Figure 1F, all data is combined for GLM decoding. We found a significant pre-to-post decrease in prediction gain specifically using the –200 to 0 ms window to predict CA1 spiking during ripples. Figure 3 builds upon these findings to examine how prediction gain changes relate to task engagement.

(2) 2b and 2c are important since they are complementary means to show the same thing, and it is important that they cross-validate each other, especially since the non-significant task active neuron difference in 2b appears to be nearly as strong as the significant difference to its left. A more holistic analysis can be done to compare these dimensions.

We appreciate this feedback. In light of this comment, as well as similar concerns raised by other reviewers regarding the binary classification of neurons as task-active or task-inactive (modulation index > 0 or < 0), we adopted a more comprehensive analytical approach. Rather than relying on an arbitrary threshold, we first examined the continuous relationship between modulation index and prediction gain change across all neurons (Figure 2C). We then divided neurons into modulation index quartiles to assess whether prediction gain changes varied across different degrees of task modulation (Figure 2D). Follow-up analyses focused on the extreme quartile comparisons that contributed to the observed effects (Figure 2E). Overall, this new approach better captured the continuous nature of task-related modulation while avoiding the inclusion of a large population of neurons with modulation indices near zero that may not meaningfully differ in task engagement. However, we were unable to replicate modulation index changes for neurons split by prediction gain score percentile and removed those figures from the manuscript. We have adjusted the text accordingly to account for this new effect.

(3) Sup vs deep neuron definition: Did the authors have any means to validate this anatomical separation using histology or otherwise? I don't believe they described anything like that, and instead use physiology to infer anatomical location. I understand anatomy-based methods may be practically impossible with tetrodes, but this limitation should at least be mentioned, and it should be explained that without something like silicon probes or histological validation, anatomy had to be inferred from physiology.

We think this is an important limitation to address and thank you for bringing this to our attention. Given our technical restraints, we only validated radial position through physiological properties. This remains an outstanding limitation of this study. We have since added text into the main discussion bringing attention to this limitation. See below:

“Lastly, our CA1 sublayer classification does come with its own caveats. Tetrode identification of CA1 sublayers is not a trivial matter. We implemented guidelines informed by past research (see methods) to help ensure we isolated CA1sup and CA1deep groups, only including neurons where we had the greatest confidence (Berndt et al., 2023; Mizuseki et al., 2011). In doing so, we excluded neurons which classification was uncertain. It is possible this excludes some meaningful populations of CA1 sublayers. Additionally, despite our approach, some cross-inclusion of sublayers may remain. Therefore, interpretations of CA1 sublayers difference should be considered with these limitations in mind. That said, the number of neurons and animals tested does provide overall confidence regarding our results. Future studies investigating this ACC to CA1 sublayer specific line of communication would benefit from the use of silicon probes or neuropixels that enable precise radial localization.”

(4) Superficial vs deep differences in firing rate ratio based on PG: there are many fewer CAdeep neurons, but in 4c, the trends appear to be the same pre-training, top PG lower than others. It seems the lack of difference in CA1deep in 4c may be due to the much lower power/n. This should be discussed or addressed.

We appreciate this feedback and since have added more recordings to address these lower Ns (CA1sup: Previous N = 71, Revisions N = 89; CA1deep: Previous N = 21, Revisions N = 61). Notably, we find that previous firing rate ratio (now called modulation index) is no longer significant with the inclusion of more neurons and is reported accordingly (Figure 2—figure supplement 1).

(5) In Figure 5, the term "firing rate ratio" is used, and it sounds the same as in previous figures, but this is a different ratio (based on modulation by opto stim, not task).

To improve clarity and avoid confusion with task-related modulation metric used across the paper, we renamed “Firing Rate Ratio" throughout the manuscript to "Modulation Index". We also relabeled the Figure 5D Y-axis as "Z-scored Firing Response" to more accurately reflect the plotted data and avoid confusion.

(6) I would like to learn more about these v-type neurons. I understand we do not yet know about their molecular or morphologic correlate, but more analysis can be done with the current data.

We thank you for this feedback. We have included further analysis into V-type properties. Namely, we performed autocorrelegrams, theta phase modulation, and burst index analyses. See Figure 6 and Figure 6—figure supplement 1.

Additionally, we performed cross-correlogram analyses to examine whether V-type interneurons exhibited consistent temporal relationships with PV interneurons or other CA1 neurons. However, V-type and PV interneurons were sparse throughout our recordings, with most sessions containing two or fewer identified interneurons, which limited our ability to perform meaningful cross-correlogram analyses. Nevertheless, we examined the available recordings but found no consistent evidence of correlated firing between interneuron classes or between V-type interneurons and CA1 pyramidal neurons.

(7) I would like more discussion of ACC-CA1 connectivity.

We have since added greater discussion of ACC-CA1 connectivity into the discussion section. See below:

“Finally, an important caveat to mention is that it remains an ongoing debate whether ACC directly projects to CA1 (Andrianova et al., 2023; Rajasethupathy et al., 2015; Shi et al., 2022). One lab reported clear monosynaptic ACC-to-CA1 connection (Rajasethupathy et al., 2015), while another lab replicated those same experiments and were unable to come to the same conclusions (Andrianova et al., 2023). Further studies report no direct connection (Shi et al., 2022). The contention in connectivity may arise from differences in targeting strategies, injection coordinates, or viruses used. Our findings reported an excitatory response in CA1 V-type interneurons in response to ACC stimulations, proposing another possibility for ACCàCA1 connectivity. Interestingly V-type interneurons responded with extremely low-latency as fast as 4.2 ms after stimulations, compatible with monosynaptic timing (Cho et al., 2013; Petreanu et al., 2007; Wang et al., 2009). If the ACC→V-Type connection was monosynaptic pathway, it could help explain discrepancies in the field, as the relative sparsity of V-type interneurons may reduce the likelihood of detecting ACC→CA1 connectivity. However, future anatomical studies are needed to conclusively determine connectivity.

Alternatively, ACC→CA1 communication may be mediated by multiple intermediate structures (Behzadi et al., 1990; Oh et al., 2014; Shi et al., 2022; Souza et al., 2022). The ACC sends monosynaptic projections to the nucleus reuniens (RE) and median raphe (MnR), both of which project directly to CA1 (Oh et al., 2014; Shi et al., 2022). The RE has a known role in contextual discrimination learning and memory specificity (Ramanathan & Maren, 2019; Ramanathan et al., 2018; Ratigan et al., 2023; Silva et al., 2021; Xu & Südhof, 2013). Interestingly, RE→CA1 activity tuned to immobility (freezing) emerges only after shocks are presented, suggesting a learning-induced modification between regions, similar to that seen in our ACC–CA1 data (Ratigan et al., 2023). As for MnR, it receives dense inputs from the ACC (Behzadi et al., 1990; Souza et al., 2022), and its projections to the CA1 are predominantly glutamatergic (Jackson et al., 2009; Senft et al., 2021; Szonyi et al., 2016). Notably, these glutamatergic MnR inputs directly target CA1 interneurons, including CCK basket cells (Miettinen & Freund, 1992; Morales & Bloom, 1997; Senft et al., 2021), while avoiding PV interneurons and pyramidal neurons (Acsady et al., 1993; Freund et al., 1990; Halasy et al., 1992; Miettinen & Freund, 1992; Papp et al., 1999; Turi et al., 2019). This connectivity suggests that the ACC may indirectly modulate CA1 activity through the MnR, potentially suppressing PV interneuron and pyramidal neuron activity via local inhibitory circuits, thereby contributing to the regulation of hippocampal oscillations and memory consolidation (Huang et al., 2022; Wang et al., 2015). Ultimately, future experiments combining pathway-specific manipulations with simultaneous recordings will be necessary to distinguish direct from polysynaptic mechanisms.”

(8) Some elements may be missing from the discussion, relating baseline functioning versus post-learning function.

We thank the reviewer for this feedback and their recommendation for possible alternate explanations. We have added these discussions into the main text. See below:

“Alternatively, ACC→CA1 communication may contribute to the homeostatic downscaling of memory-unrelated synapses during sleep. Evidence finds that slow-wave sleep is strongly linked to downscaling of non-learning related neuron activity (Gulati et al., 2017; Liu et al., 2010; Tononi & Cirelli, 2003; Tononi & Cirelli, 2006; Watson et al., 2016). Slow-wave sleep ripples in particular depotentiate memory-unrelated synapses (Gulati et al., 2017; Norimoto et al., 2018). In our study, we find that learning-related reduction in communication between ACC and CA1 were selective for task-inactive neurons. Therefore, ACC→CA1sup communication may not simply weaken following learning but rather becomes selectively disengaged from task-inactive neurons, enabling homeostatic downscaling while preserving behaviorally relevant synapses (Liu et al., 2010; Norimoto et al., 2018; Tononi & Cirelli, 2003; Tononi & Cirelli, 2006; Watson et al., 2016). Still, behavioral recruitment alone cannot account for the observed remodeling of ACC→CA1 communication, as CA1sup and CA1deep neurons did not display significant differences in task-related activity (Figure 2—figure supplemental 2). Instead, these learning-related changes of task-inactive neurons appear sublayer-specific.”

**Reviewer #2 (Public review):**

Summary:

This study uncovers an inhibitory pathway from the anterior cingulate cortex (ACC) to pyramidal cells in the superficial sublayer of hippocampal area CA1 (CA1sup). As ACC neuron spiking tends to precede hippocampal ripples, this presents the intriguing possibility that ACC inputs are selectively inhibiting particular CA1sup neurons, which could play a role in the reactivation of task-related ensembles known to take place during hippocampal ripples. Indeed, through a generalized linear model (GLM) analysis, the authors demonstrate that the ACC activity within the 200ms immediately preceding the ripple is predictive of the ripple content.

Strengths:

The biggest strength of the work is the optogenetic manipulation experiments, which convincingly demonstrate that stimulation of ACC pyramidal neurons activates an interneuron population with symmetric spike waveforms, and inhibits parvalbumin interneurons and pyramidal cells in CA1sup but not CA1deep sublayer.

An additional strength in the GLM analysis which consistently shows that ACC activity preceding the ripple is predictive of hippocampal activity during the ripple considerably more than in shuffled data for all cells and periods tested.

Weaknesses:

The major weakness of this work is that the link with learning and memory is not very well supported.

The only evidence of rebalancing and reorganization appears to be a single statistical test (the test in Figure 1f, p=0.013) demonstrating a decrease of the GLM prediction gain from pre-task sleep to post-task sleep; the same test is repeated for subsets of the data in the rest of the figures. As the idea of rebalancing and reorganization is central to the paper as currently written, exploring it through another measure, independent of the GLM prediction gain, should be expected. The notion that this pathway is suppressed in sleep following learning can be supported by demonstrating a decrease in any of the following measures: ACC spike-triggered average CA1sup responses, cross-covariances (Wierzynski et al 2009) between ACC and CA1sup cells in post-task sleep, or ripple-triggered cross-correlations (Sirota et al. 2009).

We thank the reviewer for this helpful feedback. We have added an additional analysis the reviewer pointed out to address this concern. Specifically, we performed an ACC spike‑triggered analysis. The ACC spike‑triggered average further supported the learning‑related decrease in ACC-to-CA1 activity (see Figure 1—figure supplement 1). We did not include a separate cross-covariance analysis because the ACC spike-triggered average captures essentially the same temporal relationship between ACC and CA1 activity.

The differences between task-active and task-inactive neurons are not convincing. The separation between task-active and task-inactive neurons is to divide a distribution that is far from bimodal into what appears to be two arbitrary groups. Similarly, the authors divide cells relative to their prediction gain ("Top PG" and "Bottom PG" in Figure 2c), which fails to select for the population of significantly predicted cells (relative to the shuffle). Within CA1sup cells, after learning, there is a significant decrease in the prediction gain for "task-inactive" cells but not "task-active" cells, but it is important to keep in mind that the "task-active" group contains only 24 neurons, and there was no difference between the two groups of cells ("task-active" vs "task-inactive") when directly compared.

We agree with this concern. To address this, we removed conclusion based on those arbitrary criteria instead opting for a more continuous approach. Specifically, we adopted a more comprehensive analytical approach. Rather than relying on an arbitrary threshold, we first examined the continuous relationship between modulation index and prediction gain change across all neurons (Figure 2C). We then divided neurons into modulation index quartiles to assess whether prediction gain changes varied across different degrees of task modulation (Figure 2D). Follow-up analyses focused on the extreme quartile comparisons that contributed to the observed effects (Figure 2E). Overall, this new approach better captured the continuous nature of task-related modulation while avoiding the inclusion of a large population of neurons with modulation indices near zero that may not meaningfully differ in task engagement.

Finally, it is not clear whether the identity of the pathway-responsive CA1sup neurons is fixed or whether it may change with learning. A deeper analysis into the cell pair cross-correlations or the weights of the GLM analysis may reveal whether there is a reorganization of CA1sup responses (some cells that were inhibited are no longer inhibited, and vice versa) or a dampening (the same CA1sup cells are inhibited in both cases, but the inhibition is less-pronounced in post-task sleep). The possibility of a rigid circuit dampened immediately following fear conditioning, is not discussed by the authors.

We appreciate this feedback. To address this concern without weight analysis, we examined the stability of prediction gain scores between pre- and post-training sleep. Preservation of neuronal prediction-gain rankings would suggest that learning weakens existing predictive communication while maintaining the relative contribution of individual neurons, consistent with a dampening response. In contrast, poor preservation of prediction-gain rankings would be more indicative of a reorganization of predictive relationships across the population. This led to interesting findings regarding sublayer differences: ACC→CA1sup communication is more dynamic and evolving following learning, whereas ACC→CA1deep communication remains comparatively stable (See Figure 2A&B; Figure 3 C–F).

**Reviewer #3 (Public review):**

Summary:

In this study, Hall and colleagues investigate how the coupling of activity from ACC to CA1is altered by fear learning, showing that during sleep immediately before learning, there is evidence for increased coupling of ACC activity with neurons that will subsequently be inhibited during the learning process. They go on to show that this effect seems to be mediated most by a subpopulation of neurons in the superficial layer of CA1. This fits with previous reports suggesting that these superficial neurons are key for the flexible updating of memory. The authors then go on to show that artificial activation of ACC using optogenetics results in varied effects in CA1, including a subtle decrease in activity of superficial neurons that lasts longer than the stimulus itself. Finally, the authors present some preliminary data suggesting that different interneurons may be recruited by this optogenetic stimulation in different ways and at different times.

Overall, this is an interesting paper, but much of the analysis is very preliminary, and much of the crucial data about the learning effects and alterations to cell firing are not presented clearly and fully. This is further confounded by a rather opaque description of the results and analysis in the text. Overall, there is something very interesting here, but there needs to be a substantial series of extra analyses to clearly say what this is. In many cases, more robust analysis may render the results underpowered, which could dramatically change the conclusions of the paper.

Strengths:

The authors performed difficult, dual-location recordings across a multi-day learning paradigm, which seems like it could be a really nice dataset. They delve into the circuit basis of an interesting finding regarding ACC to CA1 connectivity and how this changes before and after fear conditioning. They provide data to suggest this connectivity may be through specific and distinct subcircuits in CA1.

Weaknesses:

(1) There is essentially no information in the text or figures about what the actual learning was, how it was done, how individual animals performed, and how any of these metrics related to learning. Looking at the methods, the authors did a number of things never mentioned anywhere in the text or figures, including novel arena exposure, contextual reexposure in extinction after learning, etc. It seems that this is a very rich dataset that has not been presented at all. I would recommend at the very least:

We appreciate the reviewers’ feedback and have worked to address these concerns. See below our response.

(a) Plot all of the behavioural training data, and how each mouse relates to one another - did the mice learn? At this stage, we don't know!

We have now plotted all contextual fear conditioning behavioral data for each mouse (Figure 2—figure supplement 1). All mice exhibited high level of freezing during the contextual fear test, suggesting successful learning of the context–shock association.

(b) Explain in the text in detail exactly what was done and why, and what this tells us about the neuronal activity.

We have now added text to describe in detail the behavioral results and how that may relate to our GLM analyses. We have also more clearly detailed our experimental objectives (what was done and why) utilizing contextual fear conditioning,

“In this approach, we were able to examine ACC–CA1 communication prior, during, and after learning, enabling us to examine how this communication evolves across fear learning. Specifically, we emphasized investigation into communication changes between pre- and post-training sleep to understand whether functional connectivity undergoes learning-related reorganization.”

“Lastly, we examined whether PG scores correlated with the freezing response in mice during recall. Across all mice, freezing was significantly higher during recall than pre-shock baseline during training (Figure 2—figure supplement 2A). Overall, we found no correlation between PG and freezing (Figure 2—figure supplement 2B–D). However, there was a trend for a positive correlation (p = .07) between ΔPG and freezing percentage. An important consideration is that the uniformly high levels of freezing in mice limited behavioral variability, potentially reducing our ability to detect relationships between ACC–CA1 communication decoding and behavioral differences.”

(c) If there is variance in learning and or conditioning, does this relate to features in the analysis, such as the GLM result.

We examined this question by first investigating whether prediction gain scores in pre-training, post-training, or overall ΔPG correlated with freezing percentage. We found no significant correlation between any of the variables (Figure 2—figure supplement 1). We speculate this may be a result of a relatively robust freezing response limiting the ability for our fine-grained GLM decoding analyses to detect those differences. We have added this consideration to the main text.

(2) Along similar lines, a key metric for most of the paper is that neurons most coupled with ACC are more likely to be inhibited during training. However, there is nothing anywhere in the paper showing these data. How do neurons in general respond to contextual shocks? The methods describe this as the average firing rate during training, normalised to pre-sleep activity. This metric seems a bit coarse and may obscure really important task-relevant dynamics. Are the neurons active at specific times, are they tuned to relevant parts of the task, and do any of these features of the cell activity also relate to the coupling with ACC? Similarly, how did the authors mitigate the influence of electrical artefacts caused by the foot shock in their recordings? Again, there is a huge amount of data here that is not being described, and likely holds very valuable information about what is actually happening. The paper would really benefit from the inclusion of these data in an accessible form, such as heatmaps of spiking, how these patterns change over time, and around e.g., foot shock, etc. Also key is how these features are altered by the variability of learning across subjects.

We thank reviewer for this feedback. As pointed out, electrical artifacts caused by the footshocks prevents our ability to examine, with temporal sensitivity, neurons’ responses to footshocks. Therefore, we are left to examine activity changes across longer timescales. We acknowledge that our current modulation index analysis is a bit coarse. One reason is that our preliminary analyses using more temporally sensitive approaches did not reveal robust CA1 activity associated with specific behaviors, such as freezing or transitions between mobility and immobility. Thus, we chose a more holistic approach looking at the full CFC session to include all components that CA1 may be encoding during the training session. For example, although the pre-shock baseline period does not contain any footshock stimuli, it serves a key part in the process as mice begin to encode their environment around them. Nevertheless, we have added an additional analysis to examine how modulation changes across the pre-shock versus post-shock window (Figure 5—figure supplement 1). Although this provides greater insight into how ACC and CA1 activity changes across different dimensions in the task, further investigation utilizing casual manipulations is necessary to elucidate which phase ACC→CA1 activity is most involved.

(3) A number of the effects are presented by comparing a statistically significant effect to a non-statistically significant effect (e.g. in Figure 2b, Figure 2d, Figure 4 b,c, and others). This isn't really valid - the key test that the two groups are different is either with a direct test of the difference or an interaction term in an e.g., ANOVA test. In some places, I am not sure the same conclusions will be drawn from the data with these tests.

We want to thank the reviewer for this critical feedback. We have since added the appropriate statistical measure including linear mixed-effects models and ANOVA tests and for our analysis to avoid our previous statistical errors.

(4) To what extent is defining superficial and deep CA1 neurons solely by ripple waveform an accepted method? Of the two papers referenced for this approach, one is a 2-photon calcium imaging paper that does not do electrical recordings (as far as I am aware), and the second uses this as a descriptor after defining the positions of units on an array. It would be good to clarify how accepted this is, and also how robust this is. At the very least, some kind of metric or walkthrough in the supplement as to how this was done, and how well each cell was classified and with what confidence, or some metric of how distinct and separate the two populations were (or was it just a smudge).

We appreciate this feedback. While the Berndt 2023 paper implemented 2-photon calcium imaging, they also used tetrode classifications for radial axes in that paper which help informed our approach. Ultimately, our tetrode classification remains an outstanding limitation which we have since added to the main text (See below).

“Lastly, our CA1 sublayer classification does come with its own caveats. Tetrode identification of CA1 sublayers is not a trivial matter. We implemented guidelines informed by past research (see methods) to help ensure we isolated CA1sup and CA1deep groups, only including neurons where we had the greatest confidence (Berndt et al., 2023; Mizuseki et al., 2011). In doing so, we excluded neurons which classification was uncertain. It is possible this excludes some meaningful populations of CA1 sublayers. Additionally, despite our approach, some cross-inclusion of sublayers may remain. Therefore, interpretations of CA1 sublayers difference should be considered with these limitations in mind. That said, the number of neurons and animals tested does provide overall confidence regarding our results. Future studies investigating this ACC to CA1 sublayer specific line of communication would benefit from the use of silicon probes or neuropixels that enable precise radial localization.”

(5) In the optogenetic experiment in Figure 5, the effect on the CA1 sup neurons seems to be driven by changes in a small subpopulation of this group, with no change in the others. Related to point 2, is there anything else in the data that can pull out what these cells are? More detailed analysis of the firing of these neurons might pull out something really interesting.

We thank the reviewer for this feedback. Firstly, we want to clarify that optogenetic experiments were performed in a separate cohort of mice that did not undergo contextual fear conditioning. We have adjusted the text accordingly to make this distinction clearer. We have also added a per-animal separation of CA1 heatmap responses to ACC stimulations to demonstrate suppression is preserved across animals (Figure 5—figure supplement 3; Figure 6—figure supplement 2). Finally, our optogenetic experiments were primarily focused on understanding anatomical connectivity. Consequently, common waking behaviors (e.g., exploration and feeding) were not standardized across animals, and our analyses were therefore restricted to comparisons between slow-wave sleep and wakefulness more broadly.

(6) Related to this - a number of comparisons simply pool neurons across mice and analyse them as if independent. This is done a lot in the past, but it would be better if an approach that included the interdependence of neurons recorded from the same mouse at the same time were used (such as a hierarchical model). While this is complex, a simpler approach would just be to plot the summary data also per mouse. For example, in Figure 5, how do the neurons inhibited by ACC activation spread across the different mice? Is the level of inhibition related to how well the mice learned the CS-US association?

For dual-site analysis we have now added animal-level comparison for some key analyses (See Figure—figure supplement 1&2). As for optogenetic experiments, we have added per-animal heatmaps for ACC stimulation response (Figure 5—figure supplement 3; Figure 6—figure supplement 2).

(7) Figure 6 is interesting, but very preliminary. None of the effects are quantified, and one of the cell types is not identified. I think some proper analysis needs to be done, again across mice, to be able to draw conclusions from these data.

We thank the reviewer for this feedback. Reviewer 1 had a similar concern, and we have since added additional analyses to the revisions. Specifically, we performed autocorrelegrams, theta phase modulation analyses and a burst index analysis for V-Type interneurons. Importantly, however, these approaches still collapse neurons across mice. Given the sparse nature of V-type and PV interneurons, files often contain 2 or fewer interneurons making within-animal comparison difficult. That said, we have added supplemental figures displaying per-animal changes in response to ACC stimulations (Figure 6—figure supplement 1).

(8) Finally, in general, I felt that the way the paper was written was very hard to follow, often relying on very processed levels of analysis that were hard to relate back to the raw traces and their biological meaning. In general taking more words to really simply and fully explain each analysis, and taking the words and figures to walk through how each analysis was done and what it tells us about the neuronal data/biology would be really beneficial, especially to someone who is not an extracellular electrophysiologist or immersed in the immediate field.

We thank the reviewer for this feedback. Throughout the manuscript, we have revised the text to improve clarity in explaining our approaches and their results.

In summary, while this manuscript explores an intriguing hypothesis about pre-learning circuit dynamics, it is currently held back by insufficient clarity in behavioural analysis, data presentation, and statistical quantification. Addressing these core issues would greatly improve interpretability and confidence in the findings.

Additional comment:

For the optogenetic experiments, we reprocessed and resorted the neuronal dataset to ensure accurate cell classification. Following this re-analysis, the principal findings remained unchanged. However, we found that sublayer-specific differences in response to ACC stimulation were restricted to the first second following ACC stimulation. Consequently, we removed the previous Figure 5E, which examined firing rate changes across successive 1-sec time bins, as the additional time windows did not provide further evidence of sublayer-specific effects.

Acsady, L., Halasy, K., & Freund, T. F. (1993). Calretinin is present in non-pyramidal cells of the rat hippocampus--III. Their inputs from the median raphe and medial septal nuclei. Neuroscience, 52(4), 829-841. https://doi.org/10.1016/0306-4522(93)90532-k

Andrianova, L., Yanakieva, S., Margetts-Smith, G., Kohli, S., Brady, E. S., Aggleton, J. P., & Craig, M. T. (2023). No evidence from complementary data sources of a direct glutamatergic projection from the mouse anterior cingulate area to the hippocampal formation. eLife, 12, e77364. https://doi.org/10.7554/eLife.77364

Behzadi, G., Kalén, P., Parvopassu, F., & Wiklund, L. (1990). Afferents to the median raphe nucleus of the rat: Retrograde cholera toxin and wheat germ conjugated horseradish peroxidase tracing, and selectived-[^3^H]aspartate labelling of possible excitatory amino acid inputs. Neuroscience, 37(1), 77-100. https://doi.org/10.1016/0306-4522(90)90194-9

Berndt, M., Trusel, M., Roberts, T. F., Pfeiffer, B. E., & Volk, L. J. (2023). Bidirectional synaptic changes in deep and superficial hippocampal neurons following in vivo activity. Neuron, 111(19), 2984-2994.e2984. https://doi.org/10.1016/j.neuron.2023.08.014

Cho, J. H., Deisseroth, K., & Bolshakov, V. Y. (2013). Synaptic encoding of fear extinction in mPFC-amygdala circuits. Neuron, 80(6), 1491-1507. https://doi.org/10.1016/j.neuron.2013.09.025

Freund, T. F., Gulyas, A. I., Acsady, L., Gorcs, T., & Toth, K. (1990). Serotonergic control of the hippocampus via local inhibitory interneurons. Proc Natl Acad Sci U S A, 87(21), 8501-8505. https://doi.org/10.1073/pnas.87.21.8501

Gulati, T., Guo, L., Ramanathan, D. S., Bodepudi, A., & Ganguly, K. (2017). Neural reactivations during sleep determine network credit assignment. Nat Neurosci, 20(9), 1277-1284. https://doi.org/10.1038/nn.4601

Halasy, K., Miettinen, R., Szabat, E., & Freund, T. F. (1992). GABAergic Interneurons are the Major Postsynaptic Targets of Median Raphe Afferents in the Rat Dentate Gyrus. Eur J Neurosci, 4(2), 144-153. https://doi.org/10.1111/j.1460-9568.1992.tb00861.x

Huang, W., Ikemoto, S., & Wang, D. V. (2022). Median Raphe Nonserotonergic Neurons Modulate Hippocampal Theta Oscillations. J Neurosci, 42(10), 1987-1998. https://doi.org/10.1523/JNEUROSCI.1536-21.2022

Jackson, J., Bland, B. H., & Antle, M. C. (2009). Nonserotonergic projection neurons in the midbrain raphe nuclei contain the vesicular glutamate transporter VGLUT3. Synapse, 63(1), 31-41. https://doi.org/10.1002/syn.20581

Liu, Z.-W., Faraguna, U., Cirelli, C., Tononi, G., & Gao, X.-B. (2010). Direct Evidence for Wake-Related Increases and Sleep-Related Decreases in Synaptic Strength in Rodent Cortex. The Journal of Neuroscience, 30(25), 8671. https://doi.org/10.1523/JNEUROSCI.1409-10.2010

Miettinen, R., & Freund, T. F. (1992). Convergence and segregation of septal and median raphe inputs onto different subsets of hippocampal inhibitory interneurons. Brain Res, 594(2), 263-272. https://doi.org/10.1016/0006-8993(92)91133-y

Mizuseki, K., Diba, K., Pastalkova, E., & Buzsáki, G. (2011). Hippocampal CA1 pyramidal cells form functionally distinct sublayers. Nature Neuroscience, 14(9), 1174-1181. https://doi.org/10.1038/nn.2894

Morales, M., & Bloom, F. E. (1997). The 5-HT3 receptor is present in different subpopulations of GABAergic neurons in the rat telencephalon. J Neurosci, 17(9), 3157-3167. https://doi.org/10.1523/JNEUROSCI.17-09-03157.1997

Norimoto, H., Makino, K., Gao, M., Shikano, Y., Okamoto, K., Ishikawa, T., Sasaki, T., Hioki, H., Fujisawa, S., & Ikegaya, Y. (2018). Hippocampal ripples down-regulate synapses. Science, 359(6383), 1524-1527. https://doi.org/10.1126/science.aao0702

Oh, S. W., Harris, J. A., Ng, L., Winslow, B., Cain, N., Mihalas, S., Wang, Q., Lau, C., Kuan, L., Henry, A. M., Mortrud, M. T., Ouellette, B., Nguyen, T. N., Sorensen, S. A., Slaughterbeck, C. R., Wakeman, W., Li, Y., Feng, D., Ho, A., . . . Zeng, H. (2014). A mesoscale connectome of the mouse brain. Nature, 508(7495), 207-214. https://doi.org/10.1038/nature13186

Papp, E. C., Hajos, N., Acsady, L., & Freund, T. F. (1999). Medial septal and median raphe innervation of vasoactive intestinal polypeptide-containing interneurons in the hippocampus. Neuroscience, 90(2), 369-382. https://doi.org/10.1016/s0306-4522(98)00455-2

Petreanu, L., Huber, D., Sobczyk, A., & Svoboda, K. (2007). Channelrhodopsin-2–assisted circuit mapping of long-range callosal projections. Nature Neuroscience, 10(5), 663-668. https://doi.org/10.1038/nn1891

Rajasethupathy, P., Sankaran, S., Marshel, J. H., Kim, C. K., Ferenczi, E., Lee, S. Y., Berndt, A., Ramakrishnan, C., Jaffe, A., Lo, M., Liston, C., & Deisseroth, K. (2015). Projections from neocortex mediate top-down control of memory retrieval. Nature, 526(7575), 653-659. https://doi.org/10.1038/nature15389

Ramanathan, K. R., & Maren, S. (2019). Nucleus reuniens mediates the extinction of contextual fear conditioning. Behavioural brain research, 374, 112114. https://doi.org/10.1016/j.bbr.2019.112114

Ramanathan, K. R., Ressler, R. L., Jin, J., & Maren, S. (2018). Nucleus Reuniens Is Required for Encoding and Retrieving Precise, Hippocampal-Dependent Contextual Fear Memories in Rats. The Journal of Neuroscience, 38(46), 9925. https://doi.org/10.1523/JNEUROSCI.1429-18.2018

Ratigan, H. C., Krishnan, S., Smith, S., & Sheffield, M. E. J. (2023). A thalamic-hippocampal CA1 signal for contextual fear memory suppression, extinction, and discrimination. Nat Commun, 14(1), 6758. https://doi.org/10.1038/s41467-023-42429-6

Senft, R. A., Freret, M. E., Sturrock, N., & Dymecki, S. M. (2021). Neurochemically and Hodologically Distinct Ascending VGLUT3 versus Serotonin Subsystems Comprise the r2-Pet1 Median Raphe. J Neurosci, 41(12), 2581-2600. https://doi.org/10.1523/JNEUROSCI.1667-20.2021

Shi, W., Xue, M., Wu, F., Fan, K., Chen, Q. Y., Xu, F., Li, X. H., Bi, G. Q., Lu, J. S., & Zhuo, M. (2022). Whole-brain mapping of efferent projections of the anterior cingulate cortex in adult male mice. Mol Pain, 18, 17448069221094529. https://doi.org/10.1177/17448069221094529

Silva, B. A., Astori, S., Burns, A. M., Heiser, H., van den Heuvel, L., Santoni, G., Martinez-Reza, M. F., Sandi, C., & Gräff, J. (2021). A thalamo-amygdalar circuit underlying the extinction of remote fear memories. Nature Neuroscience, 24(7), 964-974. https://doi.org/10.1038/s41593-021-00856-y

Souza, R., Bueno, D., Lima, L. B., Muchon, M. J., Gonçalves, L., Donato, J., Jr., Shammah-Lagnado, S. J., & Metzger, M. (2022). Top-down projections of the prefrontal cortex to the ventral tegmental area, laterodorsal tegmental nucleus, and median raphe nucleus. Brain Struct Funct, 227(7), 2465-2487. https://doi.org/10.1007/s00429-022-02538-2

Szonyi, A., Mayer, M. I., Cserep, C., Takacs, V. T., Watanabe, M., Freund, T. F., & Nyiri, G. (2016). The ascending median raphe projections are mainly glutamatergic in the mouse forebrain. Brain Struct Funct, 221(2), 735-751. https://doi.org/10.1007/s00429-014-0935-1

Tononi, G., & Cirelli, C. (2003). Sleep and synaptic homeostasis: a hypothesis. Brain Research Bulletin, 62(2), 143-150. https://doi.org/10.1016/j.brainresbull.2003.09.004

Tononi, G., & Cirelli, C. (2006). Sleep function and synaptic homeostasis. Sleep Med Rev, 10(1), 49-62. https://doi.org/10.1016/j.smrv.2005.05.002

Turi, G. F., Li, W. K., Chavlis, S., Pandi, I., O'Hare, J., Priestley, J. B., Grosmark, A. D., Liao, Z., Ladow, M., Zhang, J. F., Zemelman, B. V., Poirazi, P., & Losonczy, A. (2019). Vasoactive Intestinal Polypeptide-Expressing Interneurons in the Hippocampus Support Goal-Oriented Spatial Learning. Neuron, 101(6), 1150-1165 e1158. https://doi.org/10.1016/j.neuron.2019.01.009

Wang, D. V., Yau, H.-J., Broker, C. J., Tsou, J.-H., Bonci, A., & Ikemoto, S. (2015). Mesopontine median raphe regulates hippocampal ripple oscillation and memory consolidation. Nature Neuroscience, 18(5), 728-735. https://doi.org/10.1038/nn.3998

Wang, J., Hasan, M. T., & Seung, H. S. (2009). Laser-evoked synaptic transmission in cultured hippocampal neurons expressing channelrhodopsin-2 delivered by adeno-associated virus. J Neurosci Methods, 183(2), 165-175. https://doi.org/10.1016/j.jneumeth.2009.06.024

Watson, Brendon O., Levenstein, D., Greene, J. P., Gelinas, Jennifer N., & Buzsáki, G. (2016). Network Homeostasis and State Dynamics of Neocortical Sleep. Neuron, 90(4), 839-852. https://doi.org/10.1016/j.neuron.2016.03.036

Xu, W., & Südhof, T. C. (2013). A Neural Circuit for Memory Specificity and Generalization. Science, 339(6125), 1290-1295. https://doi.org/doi:10.1126/science.1229534

**Recommendations for the authors:**

**Reviewer #1 (Recommendations for the authors):**

Suggested fixes (these correlate with the numbered points in the Weaknesses section of the Public Review):

(1) That seems like the larger finding to start with, before dissecting by Firing Activity Index. I'd first show the overall finding, then dissect it.

We thank the reviewer for this recommendation. In our manuscript, figure 1F examines the overall pre-to-post prediction gain changes prior to any neuron separation. Further separation of neurons’ characteristics and classification occurs in subsequent figures.

(2) The overall picture painted by Figure 2 suggests a correlation analysis should be carried out to search for a general property: prediction score gain vs firing activity index. Are those two variables considered significant by Pearson correlation? Did the authors try that and it didn't work, so they did these analyses? If there is no significant correlation, what does a more detailed look at those two variables on an x-y plot teach us? I suggest considering showing such a plot to readers, at least in a Supplement.

We thank the reviewer for this feedback and have incorporated this approach in our revised manuscript. Specifically, we performed a correlation analysis between prediction gain change and modulation index (formerly called firing activity index). We uncovered a significant positive correlation between variables, suggesting that task engagement modifies ACC→CA1 communication (Figure 2C).

(3) (No additional comments).

(4) This weakness may be able to be addressed by doing a correlation of depth (LFP amplitude of sharp wave) versus the firing rate ratio. For example, the threshold used for deep may have been such that it reduced the number of detected deep neurons, but if a general relationship between depth and degree of FRR is found, it can remove issues from this difference in statistical power.

We thank the reviewer for this comment. We were unable to perform a reliable link between correlation depth and modulation index. LFP amplitude can vary substantially between tetrodes due to differences in electrode impedance, placement, and recording conditions, making direct comparisons across animals difficult. While within-animal analyses could largely circumvent these issues, many recording sessions did not include tetrodes spanning the full superficial-to-deep CA1 axis, preventing a reliable assessment of this relationship.

(5) I would give it a different name - "opto-modulation index" or "opto firing rate ratio" perhaps. This would make it clear that you are not measuring task-based modulation of firing.

We have since modified our wording to improve clarity. Specifically, we renamed “Firing Rate Ratio" throughout the manuscript to "Modulation Index". We also relabeled the Figure 5D Y-axis as "Z-scored Firing Response" to more accurately reflect the plotted data and avoid confusion

(6) Specifically: can the post-opto lag of v-type versus wide-waveform and PV-type neurons be analyzed? Are the V-type neurons increasing firing before the others decrease? What about cross correlograms between v-type and pyramidal neurons, either at baseline or post-stim?

We appreciate this feedback. We have added a figure showing differences in response lags to the optostimulation. We demonstrate that V-Type interneurons clearly fire prior to PV and pyramidal cells (Figure 6—figure supplement 1E&F). Additionally, we performed cross-correlogram analyses to examine whether V-type interneurons exhibited consistent temporal relationships with PV interneurons or other CA1 neurons. However, V-type and PV interneurons were sparse throughout our recordings, with most sessions containing two or fewer identified interneurons, which limited our ability to perform meaningful cross-correlation analyses. Nevertheless, we examined the available recordings but found no consistent evidence of correlated firing between interneuron classes or between V-type and pyramidal neurons.

(7) Can the authors discuss the candidate pathways for connectivity from ACC to CA1?

We thank the reviewer for this feedback. We have since added discussions on ACC-to-CA1 connectivity and discussed possible relay brain regions between ACC and CA1.

(8) There is mounting evidence about the role of sleep oscillatory events playing homeostatic roles, not only memory-based. The authors bring this up, but do not offer it as an explanation for their findings, but I believe they probably should. For example, Norimoto et al 2018 cited by the authors. Also, Gulati/Gunguly et al 2017 Nature Neuroscience suggests downscaling as a default NonREM activity. Gulati and also Roux/Buzsaki NatNeuro 2017 show that certain privileged or tagged neurons can be protected from this. This therefore reflects that default activity in nonREM may have a homeostatic role, but then learning may alter that default. I believe this should be discussed as a possible reason for the dissociation between ACC and CA1, the authors observe after CFC.

In more detail, the authors state that CFC worsened ACC ability to predict ripple spike rate vectors. The authors suggest this may reflect "worsened" communication from ACC to HPC. It could also reflect a SHIFT (not worsening) in the information state of the hippocampus, where ripples reflect novel information and/or information coming from other brain regions. Essentially, the novel information may out-compete usual information flows. For example, ACC may be a default "feeder" into ripples (for example as part of default mode network) when there was no recent highly salient information, but under non-default conditions such as after CFC, ripple content may be fed from other sources (be they internal or external to the HPC). I believe this should be discussed.

For example, were CA1 sup task inactive neurons basically DMN-active neurons? Figure 2 shows neurons with the highest pre-training ACC prediction were the ones that dropped the most in training - again suggesting these neurons may be tuned to internal or default dynamics rather than CFC (or other novel experiences).

This shift from a default communication mode to a more experience-based one should probably be discussed as an alternative explanation, rather than simply "worsening" of communication.

We thank the reviewer for this feedback and their recommendation for possible alternate explanations. We have incorporated many of the listed citations and ideas they discussed into our discussion section proposing homeostatic downscaling and a shift in the default mode network as possible explanations for our results.

Minor Weaknesses:

(1) Introduction Line 52: "during replays" should probably be "during replay events".

Changed.

(2) Introduction Line 76: "how communications" should be "how communication"

Changed.

(3) 200-0, 400-200, 600-400 time bins are a bit unclear in Figure 1f. Are they really negative times, rather than positive? Perhaps negative signs could be put into the legend of 1f, or the time windows can be shown on the left side of 1e. Or potentially 1f could use the same -0.6, -0.4, etc as 1e so readers understand they are linked (if I understand correctly).

They are negative in the sense the occur before the ripple event. Figure 1e now displays the negative signs.

(4) I don't believe the methods describe how many tetrodes are put into the ACC. It would seem this should be put in the ACC portion of the "Stereotaxic surgery" section.

We now clearly explain the number of tetrodes (8) in the "Stereotaxic surgery" section.

(5) Results line 125: "learning induced" should be "learning-induced".

Changed.

(6) In terms of display, deep and sup are swapped in the various figures in terms of which is shown first/second (at least for readers assuming left is first). I suggest putting deep first or sup first in all figures. To me, sup first seems more natural, but homogeneity seems best regardless. This will help readers easily track results.

We adjusted the figures so that superficial is typically displayed first with some exceptions. For example, in Figure 3A, CA1deep is shown first to preserve the anatomical (dorsal-ventral) relationship.

(7) Results line 178: "optogenetics stimulation" should be "optogenetic stimulation".

Changed.

(8) Results line 180: "upon stimulations" should be "upon stimulation".

Changed.

(9) Results line 180: "to different capacities" could be "to different degrees" or "in different manners".

Changed.

**Reviewer #2 (Recommendations for the authors):**

The sleep scoring procedure is not described clearly. The text references delta waves and ripple oscillations, but the accompanying citation (Wang et al. 2015) does not use such a procedure. Since the post-task rest sessions are called "sleep sessions", there is some confusion about whether the data was restricted to slow wave sleep or not. If data from each sleep session were taken without restricting to actual sleep, that would be problematic because animals may be less likely to sleep immediately following fear conditioning, which could introduce some sleep/wake bias into the comparisons. In particular, the relationship between cortex and ripple activity has been reported to dramatically change between awake and sleep states (Tang & Jadhav, 2019). I am not including this point in the public review in case the data was in fact restricted for sleep, and it simply needs to be clarified in the text.

Thank you for this feedback. The recordings were in fact restricted to sleep. We have added text to the manuscript to make this clearer. Moreover, we added further discussion on how sleep was calculated.

There is a puzzling paragraph in the discussion, arguing that "Here, we add to this understanding with CA1sup neurons having a diminished role in fear memory formation". Sparse task-related activity in CA1sup does not imply that CA1sup is not involved in memory. Indeed, while the median of CA1sup neurons' firing rate ratio was below zero, there is a substantial proportion of neurons that are recruited, and these could be extremely important for memory. Most studies on reactivation and replay would only concentrate on cells sufficiently active in the task, and observe whether these patterns of activity are enhanced in post-task sleep.

We thank the reviewer for this feedback and have removed the text claiming sublayer difference in fear conditioning.

If the ACC is indeed inhibiting the CA1sup pyramidal cells through V-type interneurons, then one would expect the average GLM weights predicting the activity of those best-predicted CA1sup cells to be negative. If that is true, that could nicely tie the prediction effect to the optogenetic results, demonstrating that ACC's relationship to pyramidal cells is inhibitory in natural conditions as well.

We thank the reviewer for this suggestion. Unfortunately, our primary GLM analysis did not properly save weight coefficients to perform such analyses. To address this as closely as possible, we performed a preliminary analysis using a modified version of our GLM to examine whether the coefficients predicting CA1sup pyramidal neuron activity exhibited a bias toward negative weights. While this modified analysis did not generate coefficients directly comparable to the prediction gain values reported in the manuscript, it allowed us to assess whether an overall difference in coefficient sign was evident between CA1 sublayers. We found no significant bias toward negative coefficients and no clear differences between CA1sup and CA1deep neurons. Although this result does not provide additional support for an inhibitory relationship under natural conditions, it does not necessarily contradict our optogenetic findings. GLM coefficients quantify statistical dependencies between neural activities and reflect not only direct interactions but also indirect network effects, shared inputs, and the model structure. Consequently, the sign of a GLM coefficient should not be interpreted as a direct measure of whether the underlying synaptic relationship is excitatory or inhibitory.

Figure 1f is strangely missing comparisons for positive delays. If such windows were to be included and if the reactivation gain is lower for them, that could really drive home the point that communication takes place in the ACC->CA1 direction more than in the CA1->ACC direction.

Our goal of this study was to examine how incoming information from the cortex may differentially drive CA1 sublayer activity. While we think examining the reverse direction offers a compelling future direction, it was beyond the scope of our present manuscript.

There is some confusion about the N-s. There's a total of 190 CA1 neurons (Figure 2a legend). 21 of them are deep, and 77 are sup (Figure 3b legend), so presumably 92 would be neither. The legend of Figure 4b agrees with this: 24 task-active CA1sup and 53 task-inactive CA1sup cells, while in CA1deep, there were 14 task-active and 7 task-inactive cells, but in Figure 3c, there is a comparison of N=24 CA1deep cells and n=94 CA1sup cells (so 72 neither).

For one dual-site animal, the CFC recording file was corrupted, while the pre- and post-training recordings remained intact. As a result, this animal was included only in the GLM analyses, leading to slight differences in sample size across analyses. We have clarified these sample sizes in the revised manuscript and highlighted this discrepancy in the Methods section.

There appears to be a typo on line 213, the reference should be to Figure 6f.

Changed.

**Reviewer #3 (Recommendations for the authors):**

To what extent do the authors think that this is learning dependent, as opposed to stress dependent? Not that I want an experiment here, but it is important to note that both of these regions are very much involved in stress responses. Do you think you would get the same result with a purely appetitive learning paradigm? Or is this specific to stress? It might be nice to add this to the discussion.

We thank the reviewer for this raising this point of discussion. Future experiments utilizing appetitive behavioral tasks could help address these important questions. While we have avoided speculating too much, we agree it is valuable to acknowledge this possibility. Accordingly, we have added a brief discussion point to at least call attention to this possibility for the reader.

“Another caveat to mention is that both the ACC and CA1 are involved in the stress response (Kim et al., 2015; Lamotte et al., 2021). Future experiments utilizing appetitive learning paradigms, rather than the aversive contextual fear conditioning used here, will help disentangle learning-related remodeling of ACC→CA1 communication from changes driven by stress.”

There are a number of typos that confuse the message - for example, in the abstract, the authors say that ACC suppresses superficial CA1 interneurons. This seems most likely an error - I think the authors mean superficial CA1 neurons? Or PV interneurons? Similar errors exist throughout, as well as odd combinations of bold and italics across and within words, etc. Overall, especially in consideration of my final main point above regarding clarity of the text, it would be good to have a proper proofread to make sure the text is as clear as possible

We thank the reviewer for identifying these issues. The specific error in the abstract has been corrected, and we have since carefully proofread the revised manuscript.
